# Signaling Pathway Inhibitors, miRNA, and Nanocarrier-Based Pharmacotherapeutics for the Treatment of Lung Cancer: A Review

**DOI:** 10.3390/pharmaceutics13122120

**Published:** 2021-12-08

**Authors:** Shadab Md, Nabil A. Alhakamy, Shahid Karim, Gamal A Gabr, Mohammad Kashif Iqubal, Samar S. A. Murshid

**Affiliations:** 1 Department of Pharmaceutics, Faculty of Pharmacy, King Abdulaziz University, Jeddah 21589, Saudi Arabia; nalhakamy@kau.edu.sa; 2 Center of Excellence for Drug Research & Pharmaceutical Industries, King Abdulaziz University, Jeddah 21589, Saudi Arabia; 3 Mohamed Saeed Tamer Chair for Pharmaceutical Industries, King Abdulaziz University, Jeddah 21589, Saudi Arabia; 4 Department of Pharmacology, Faculty of Medicine, King Abdulaziz University, Jeddah 21589, Saudi Arabia; skaled@kau.edu.sa; 5 Department of Pharmacology and Toxicology, College of Pharmacy, Prince Satam Bin Abdulaziz University, Al-Kharj 16278, Saudi Arabia; g.gabr@psau.edu.sa; 6 Department of Pharmaceutics, School of Pharmaceutical Education and Research, Jamia Hamdard, New Delhi 110062, India; mkashifiqubal@ymail.com; 7 Sentiss Research Centre, Product Development Department, Sentiss Pharma Pvt Ltd., Gurugram 122001, India; 8 Department of Natural Products and Alternative Medicine, Faculty of Pharmacy, King Abdulaziz University, Jeddah 21589, Saudi Arabia; samurshid@kau.edu.sa

**Keywords:** lung cancer, angiogenesis, apoptosis, miRNA, oncomiRs

## Abstract

Lung cancer is one of the most commonly diagnosed cancers and is responsible for a large number of deaths worldwide. The pathogenic mechanism of lung cancer is complex and multifactorial in origin. Thus, various signaling pathways as targets for therapy are being examined, and many new drugs are in the pipeline. However, both conventional and target-based drugs have been reported to present significant adverse effects, and both types of drugs can affect the clinical outcome in addition to patient quality of life. Recently, miRNA has been identified as a promising target for lung cancer treatment. Therefore, miRNA mimics, oncomiRs, or miRNA suppressors have been developed and studied for possible anticancer effects. However, these miRNAs also suffer from the limitations of low stability, biodegradation, thermal instability, and other issues. Thus, nanocarrier-based drug delivery for the chemotherapeutic drug delivery in addition to miRNA-based systems have been developed so that existing limitations can be resolved, and enhanced therapeutic outcomes can be achieved. Thus, this review discusses lung cancer’s molecular mechanism, currently approved drugs, and their adverse effects. We also discuss miRNA biosynthesis and pathogenetic role, highlight pre-clinical and clinical evidence for use of miRNA in cancer therapy, and discussed limitations of this therapy. Furthermore, nanocarrier-based drug delivery systems to deliver chemotherapeutic drugs and miRNAs are described in detail. In brief, the present review describes the mechanism and up-to-date possible therapeutic approaches for lung cancer treatment and emphasizes future prospects to bring these novel approaches from bench to bedside.

## 1. Introduction

According to World Health Organization (WHO), lung cancer (LC) is a major cause of death and is the second most commonly diagnosed cancer worldwide. According to the published report in 2020, 2.2 million (11.44%) new lung cancer cases were diagnosed, and 1.8 million (18%) deaths were reported worldwide. It is further predicted that by the end of 2035, the mortality rate due to LC may exceed 3 million [1,2]. LC is the leading cause of death in men, whereas it is the third leading cause of death in women following breast and colon cancer [2]. As per published report in 2021, in 112 countries, prostate cancer has been commonly diagnosed as the leading cause of death, followed by LC in 93 countries and colorectal cancer in 11 countries [2]. Another challenge for LC is its survival rate. The average survival rate of patients was reported to be 10–20% with the highest survival rates found in Japan, Israel, and Korea (33%, 27%, and 25%, respectively) [2]. Among various risk factors, smoking is one of the factors that is responsible for the pathogenesis of LC. More than 80% of deaths among confirmed cases of LC are caused by smoking, while 4.7–14% are caused by inhaling particles less than 2.5 µM (PM_2.5_) [3]. It has also been demonstrated that passive smoking contributes to one-third of the total cases. Among various airborne particulates and gases, asbestos (a carcinogen) is primarily responsible for LC, and when smokers are exposed to it, the chance of developing LC increases significantly [4,5,6,7,8]. Radon is a gas produced by radium 226 that becomes trapped in buildings with poor ventilation. Radon emits an alpha particle, and hence, exposure to this gas initiates an LC cascade. Aside from the factors mentioned above, previous exposure to heavy metals, such as nickel, chromium, aromatic hydrocarbons, ether, and α1–antitrypsin deficiency have been identified as major causes of LC [9,10,11]. However, with the understanding of the cellular and molecular etiology, it was found that miRNA deregulation significantly contributes in the etiology of LC. Based on the histological findings, LC is subdivided into small cell and non-small cell LC (SCLC and NSCLC). NSCLC represents approximately 85–90% of cases, and SCLC represents 10–15% of cases [12]. At present, chemotherapy, radiotherapy, and surgery either alone or in combination are the available therapeutic regimens. Among the three available options, chemotherapy is extensively used. However, the use of chemotherapy or targeted anticancer drugs is associated with multiple challenges, such as unintended side effects, multi-organ toxicities, and drug resistance [13]. Chemotherapeutic drugs are non-selective in nature, which means, when administered, they damage healthy or normal cells in addition to cancerous cells, resulting in homeostatic alterations. To overcome these challenges, nanocarrier-based targeted drug delivery and miRNA-based pharmacotherapeutic regimens with site-specific mechanisms of action have been developed. Thus, in this review, we discuss the mechanistic pathogenesis of lung cancer, available pharmacotherapeutic regimens, their challenges and limitations, promising roles of various nano-encapsulated natural/synthetic drugs, and the emerging role of miRNA in the management and treatment of LC.

## 2. Signaling Pathways and Targeted Therapy in LC

The pathogenesis of LC is complex and multifactorial in origin. Its pathogenesis is mediated by several cellular and molecular signaling pathways, and selective targeting of these pathways is now being considered as a novel and targeted therapeutic approach for treating LC [13]. Signaling pathways are either stimulated by the pro-oncogenes or inhibited by anti-oncogenes, resulting in tumor proliferation, migration, angiogenesis, and apoptotic escape [14]. Thus, inhibition of pro-oncogenes and stimulation of anti-oncogene-related signaling pathways are emerging targets for various pharmacotherapeutics.

### 2.1. Epidermal Growth Factor (EGFR) Receptor Deregulation and EGFR Inhibitors in LC

**Figure 1:**
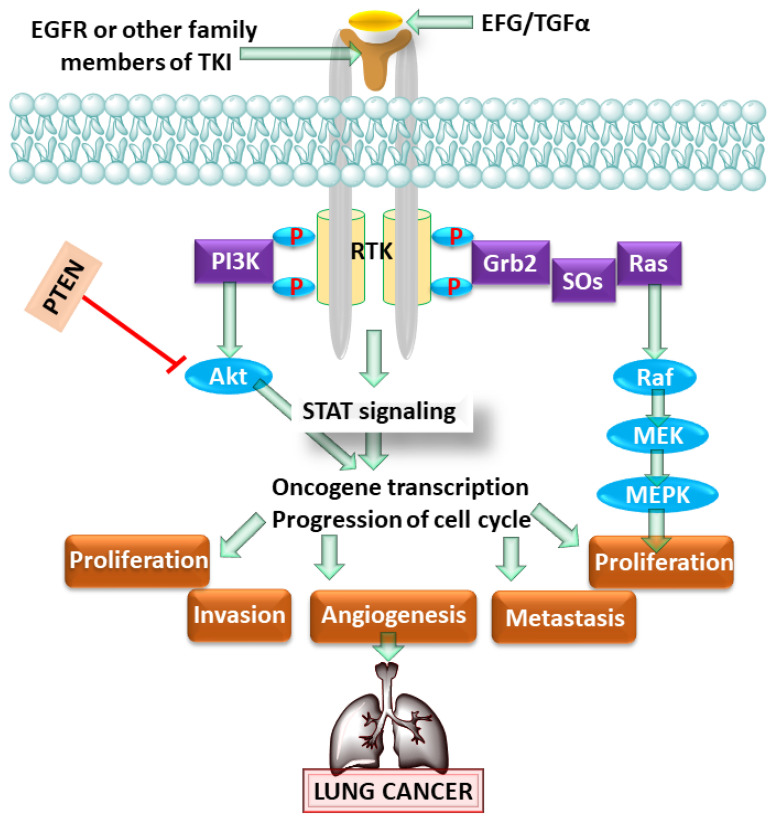
Showing the role of the epidermal growth factor receptor (EGFR) pathway in lung cancer. epidermal growth factor (EGF), transforming growth factor (TGFα), Growth factor receptor-bound protein 2 (Grb2), son of sevenless (SOs), Mitogen-activated protein kinase (MAPK) and signal transducer and activator of transcription (STAT).

Altered cellular proliferation is considered as one of the primary causes of LC initiation and progression, in which normal or healthy cells are transformed into malignant cells [15]. Numerous published reports have shown the pathogenetic role of the epidermal growth factor receptor (EGFR) in tumor initiation and progression. EGFR is a member of the tyrosine kinase receptor (RTK) family, as shown in Figure 1 [16]. In clinical studies, 43–89% of cases of NSCLC were found to be related to EGFR mutations [17].

Currently, erlotinib and gefitinib are the two drugs that cause functional inactivation of the EGFR intracellular domain and are used to treat lung cancer [14]. Cetuximab and bevacizumab are two potent monoclonal antibodies (mAb) and act as EGFR blockers [14]. When cetuximab was used in combination with radiotherapy, a synergistic anticancer effect was observed. When cisplatin and vinorelbine were used in combination with cetuximab, a significant improvement in patient survival rate was found [18]. Additionally, erlotinib and gefitinib were shown to penetrate the lung’s tumor cells more potentially than cetuximab [19]. A Phase III clinical trial (OPTIMAL) was conducted to compare the efficacy and tolerability of erlotinib against standard chemotherapy. Among patients with EGFR mutation, specifically with EGFR 19 deletion or an EGFR L858R point mutation, the results revealed significant clinical outcomes with minimal side effects [20]. TORCH, another Phase III trial, was conducted to determine the efficacy of erlotinib and cisplatin–gemcitabine among the patient with advanced NSCLC, and the outcome showed superior efficacy of erlotinib [21]. Similarly, the Phase III TITAN trial was conducted to check the efficacy of erlotinib against docetaxel among the patients of recurrent NSCLC. The result showed no significant difference in terms of clinical outcome, effectiveness, and safety of erlotinib versus chemotherapy in second-line treatment of patients with advanced, non-small-cell lung cancer with poor prognosis [22]. The outcome of the Phase III trial (INFORM), in which gefitinib was studied as maintenance therapy among patients with advanced NSCLC, showed significantly beneficial clinical outcomes [23]. Likewise, cetuximab (an anti-EGFR mAb) was also tested in a Phase III clinical trial (FLEX), and the outcome of this trial showed significant clinical benefit and reduced EGFR expression [24].

### 2.2. Vascular Endothelial Growth Factor (VEGF) Receptor Deregulation and VEGF Inhibitors in LC

**Figure 2:**
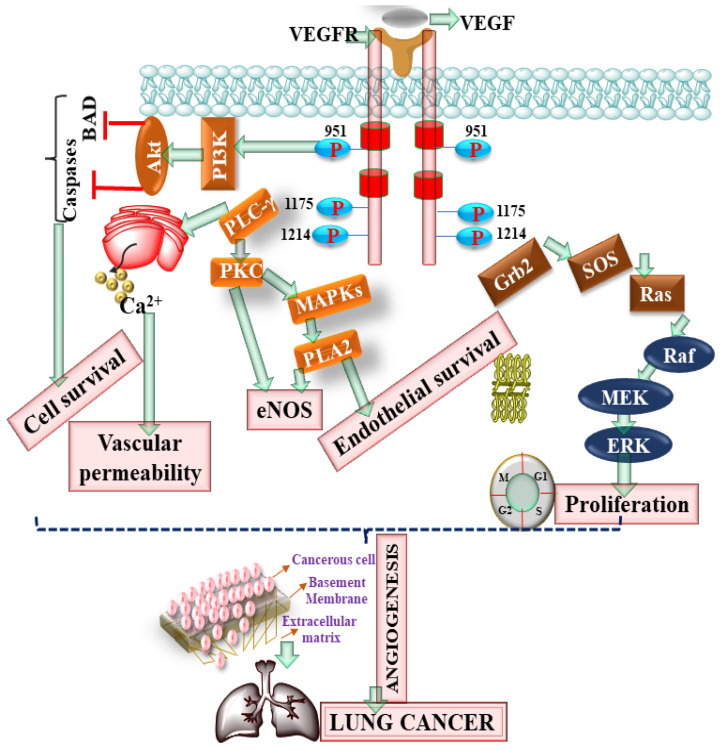
Showing the role of the vascular endothelial growth factor receptor (VEGFR) pathway in lung cancer. Protein kinase B (Akt), phosphoinositide 3-kinases (PI3K), (PLC), phospholipase C (PLC), protein kinase C (PKC), Phospholipase A2 (PLA2), extracellular-signal-regulated kinase (ERK), and endothelial nitric oxide synthase (eNOS).

Angiogenesis is the process of development of vasculature from coexisting blood vessels in response to normal physiological processes such as growth, reproduction, and development of various organs. Angiogenesis is strictly controlled under normal physiological conditions and occurs only for a limited period [25]. However, during carcinogenesis, the physiological balance becomes disrupted, and uncontrolled angiogenesis occurs as shown in Figure 2. During angiogenesis, endothelial and cancerous cells release proangiogenic factors, such as vascular endothelial growth factor, fibroblast growth factor, and transforming growth factor-beta (VEGF, FGF, and TGF-β, respectively) that regulate angiogenesis in association with other signaling molecules [25].

Pazopanib is a tyrosine kinase inhibitor (TKI) that inhibits the growth of tumor cells and the process of angiogenesis [26]. A Phase II clinical trial was conducted to determine the efficacy of pazopanib among patients with NSCLC. The study’s outcome showed excellent tolerability and reduction in the tumor volume [26]. Vandetanib is also a TKI and inhibits EGFR and VEGF. A Phase III clinical trial (ZEPHYR) was conducted to determine the efficacy and tolerability among the patients with NSCLC. However, unfortunately, the outcome of this trial showed non-significant clinical outcomes and some serious side effects [27]. Bevacizumab is a mAb and is the first approved drug to inhibit angiogenesis via selectively targeting VEGF among NSCLC patients [28]. In a Phase III clinical trial (ECOG 4599), the combined use of bevacizumab with carboplatin and paclitaxel showed a significant clinical outcome along with prominent hypertension [29]. In another Phase III clinical trial (BeTa), the combination of bevacizumab and erlotinib was studied among NSCLC patients [30]. However, in another Phase III clinical trial, bevacizumab used in combination with cisplatin and gemcitabine demonstrated a significant improvement in survival time [31]. Apart from these three drugs, motesanib, axitinib and BIBF1120 are currently being evaluated in different clinical trial phases [32].

### 2.3. PI3K/AKT/mTOR Signaling Pathway and PI3K/AKT/mTOR Inhibitors in LC

**Figure 3:**
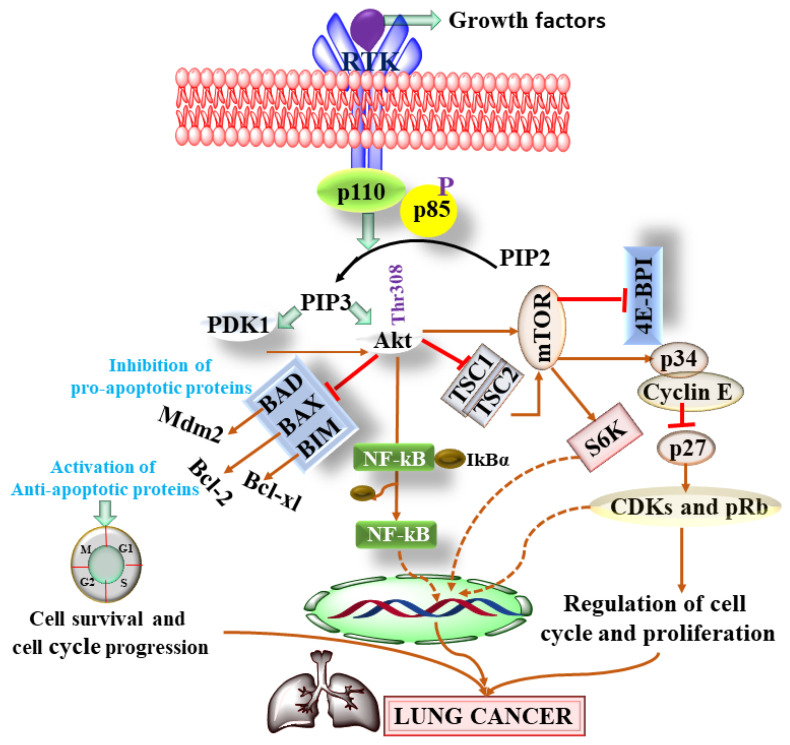
Showing the role of PI3K/Akt pathway in lung cancer. Pyruvate dehydrogenase kinases (PDKs), Phosphatidylinositol 4,5-bisphosphate (PIP), mammalian target of rapamycin (mTOR), eukaryotic translation initiation factor 4E-binding protein 1 (4E-BP1), TSC Complex Subunit 1 (TSC1), cyclin-dependent kinase (CDK), pRB (retinoblastoma protein), mouse double minute 2 (Mdm2), nuclear factor kappa-light-chain-enhancer of activated B cells (NF-kB) and nuclear factor of kappa light polypeptide gene enhancer in B-cells inhibitor, alpha (IKBα).

Phosphoinositide 3-kinase (PI3K) is one of the important members of the lipid kinase family. HER2 and IGF receptors are considered upstream regulators of PI3K [33]. Under stressed conditions or after ligand binding, p11 regulates phosphorylation of PIP2 to PIP3 and results in the activation of protein kinase B (AKT) [33]. Once AKT is activated, it becomes separated from the surface of the cell membrane and modulates various downstream signaling pathways as shown in Figure 3. Mammalian target of rapamycin (mTOR) is one of the important and extensively studied serine/threonine kinases and is present in the form of mTOR1 and mTOR2, as shown in Figure 3 [33].

Considering the role of the PI3K/AKT/mTOR signaling pathway in LC, it was found that this pathway is significantly activated (50–73%) in NSCLC [34,35]. Apart from PI3K/AKT’s proven role in LC, the study also showed the involvement of the mTOR pathway and reported 30% mutated mTOR among 188 patients [36].

The pan-PI3K inhibitors are a class of drugs that bind to p110 and abrogate PI3K activation. The PI3K inhibitors, GDC 0941, 0032, and 0973, PX-866, BKM 120, XL-147, and BAY 80-6946 are some of the recently developed pan-PI3K inhibitors for LC patients in various clinical studies [36]. Apart from the pan-PI3K inhibitors, AKT inhibitors have also been developed to eventually stop LC tumor survival and mitogenic properties. GDC 0068, NVP-BKM120, GSK 2141795, MK2206, perifosine A-443654, and GSK690693 are AKT inhibitors that have shown significant antitumor effects in LC patients [36]. Additionally, when the AKT inhibitor, MK-2206, was used with other chemotherapeutic drugs, such as docetaxel, doxorubicin, and erlotinib, the combinations showed significant synergistic anticancer effects [36]. Everolimus, ridaforolimus, deforolimus, and temsirolimus are mTOR1 inhibitors and are approved for treating various types of cancer, including LC. The mTOR1 inhibitors, such as everolimus, were tested in combination with gefitinib, docetaxel, and sorafenib, and the outcome of these studies showed synergistic antitumor effects [37]. Apart from mTOR1 inhibitors, dual mTOR1/2 inhibitors have also been developed and tested in various clinical studies. OSI-027 and INK128 are two mTOR1/2 inhibitors that additionally show inhibitory actions toward VEGF and heat inducible factor (HIF-1α) [38]. Interestingly, not only dual mTOR1/2 inhibitors, but also PI3K/mTOR inhibitors, act as inhibitors of both PI3K and mTOR [36]. XL765, BEZ235, PF-04691502, NVP-BEZ235, BGT226, PI-103, and GDC-0980 are some of the tested dual-acting PI3K/mTOR inhibitors among patients with LC [36].

### 2.4. p53, Bax/Bcl-2, Fas, and p16INK4/Cyclin D1/Rb Pathway Dysfunction and Their Inhibitors in LC

p53 is one of the most extensively explored tumor suppressor genes; it acts as a gatekeeper and maintains genetic stability. p53 senses stress, mutagenic action, damage to DNA, hypoxia, and activation of pro-oncogenes [39]. In LC, the p53 mutation has been extensively studied [39]. Exposure to PM_2.5_, cigarette smoke, and other carcinogens cause transverse mutation, for example, change of TA to GT and GC to TA (G–A) transitions, which are responsible for LC [40]. Various downstream signaling molecules, such as B-cell lymphoma 2 (Bcl-2), which is anti-apoptotic and downregulated, Bcl-2-associated X protein (Bax), which is pro-apoptotic and upregulated, Fas, tumor necrosis factor receptor-like apoptosis-inducing ligand (TRAIL), and death receptor 5 (TRAIL-DR5; upregulated) are under the control of p53 and act to modulate apoptosis in LC [41].

One of the important features of cancer cells is evasion of apoptosis. Apoptotic evasion is an important phenomenon that promotes both tumor growth and proliferation [42]. Bcl-2 and Bax are two apoptotic proteins involved in mitochondrial-mediated apoptosis [42]. In LC, overexpression of Bax and deficiency of Bcl-2 proteins have been reported [43]. Death receptor-mediated apoptosis is another mechanism involved in the antitumor effect [44]. When FasL binds to its receptor, the subsequent signaling pathway is activated and causes apoptosis via caspase-8. In LC, the Fas receptor was found to be downregulated, suggesting apoptotic evasion [44].

ABT-737 and Bcl-2 antisense oligonucleotides were developed to trigger apoptosis in the case of NSCLC [45]. Considering the role of TRAIL in LC, rhTRAIL (AMG 951), Mapatumumab (anti-TRAIL-R1 mAb), and AMG 655 have been developed to target the death receptor in the lungs [32]. These drugs are currently under different phases of clinical trials and are in the pipeline for approval. Apart from these pipeline drugs, several small molecules, such as sorafenib (RAF/MEK/ERK inhibitor), AZD6244 (mitogen-activated extracellular signal-regulated kinase (MEK) inhibitor), and enzastaurin (serine/threonine inhibitor) are being tested either alone or in combination with other anticancer drugs among the patient with LC [32].

## 3. Limitations of the Approved and Pipeline Drugs of Lungs Cancer

Currently, various anticancer drugs have been approved for the treatment and management of LC. However, most of the conventional and signaling pathway-specific drugs exhibit significant long- and short-term adverse effects, such as cardiotoxicity, hepatotoxicity, nephrotoxicity, rashes, and others [46]. Apart from these significant adverse effects, these approved drugs also cause drug resistance, leading to a poor rate of patient survival and low quality of life. In one of the clinical studies, cisplatin and etoposide were used for the treatment of LC, and thrombocytopenia, leukopenia, and neutropenia were observed [47]. Additionally, most chemotherapeutics are administered via the oral route. These drugs possess poor solubility, low bioavailability, and permeability, and cause GI irritation. To overcome this problem, inhalation-based drug delivery was used, but unfortunately, the direct exposure of the drug to the lungs caused significant pulmonary toxicity. Details of the mechanism of action, year of approval, and adverse effects are shown in Table 1.

## 4. Nanocarrier-Based Targeted Drug Delivery in LC

**Figure 4:**
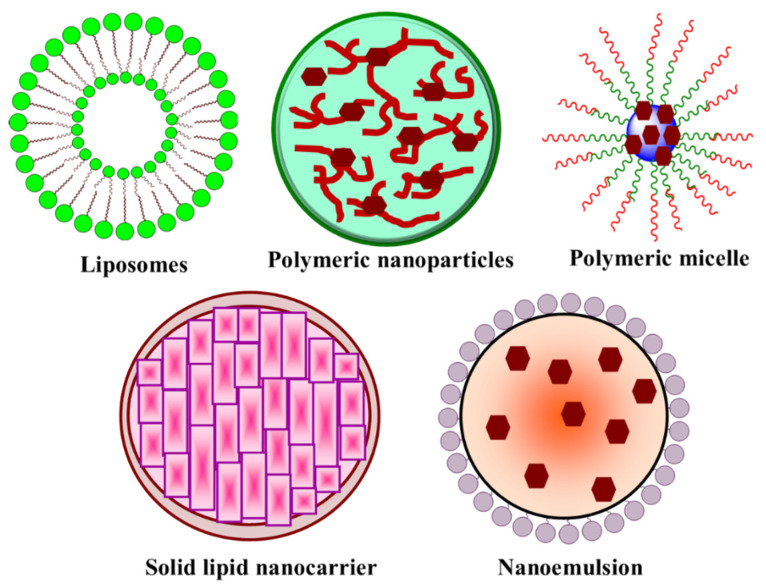
Showing various nanocarriers used for drug delivery in lung cancer.

As far as conventional chemotherapeutic drugs are concerned, no doubt these agents are potent and effective therapeutic moieties. Still, non-specificity, adverse effects, and poor pharmacokinetic profiles are limiting factors for their use [50]. Thus, in recent years, various nanocarriers, such as liposomes, nanoemulsions, polymeric nanoparticles, and polymeric micelles have been fabricated, as shown in Figure 4 [51]. This development is in response to problems caused by conventional drugs can be overcome and targeted drug delivery, enhanced pharmacological effect, and mitigation of adverse effects can be achieved [51]. These nanocarrier systems vary greatly in shapes, sizes, and surface charges. One of the advantages of these nanocarriers is the delivery of various drugs without using any toxic excipients [52]. Concerning targeted drug delivery in the lung, nanocarriers easily cross the various barriers and prolong the drug residence time in the tumor environment via escape from mucociliary clearance and phagocytosis in lung cells [53]. Currently, various nanocarriers are being studied in the clinical and preclinical setups, and some of them have entered clinical trials.

### 4.1. Polymeric Nanoparticles

Polymeric nanoparticles (PNPs) are mainly prepared from either natural or synthetic polymers. Based on the surface charge, polymeric PNPs are classified as cationic or anionic. Cationic PNPs are positively charged because of the presence of primary, secondary or tertiary amines, and subdivided as natural or synthetic PNPs. Cationic polymers are less toxic, possess improved encapsulation efficacy and offers controlled release. Additionally, cationic polymers can encapsulate hydrophobic drugs which are otherwise impermeable to the cell membrane and DNA. Some of the commonly studied polymers used in the fabrication of PNPs consist of chitosan, cyanoacrylates, poly (lactic-co-glycolic) acid (PLGA), gelatin, poly alkyl-, poly (lactic acid) (PLA), albumin, and polycaprolactone. These polymers are biodegradable and offer a controlled release pattern. Currently used intravenous anticancer drugs for lung cancer treatment are not feasible for patients as their use has been reported to cause systemic toxicities, pain, and discomfort. Orally-used anticancer drugs suffer from the lack of significant clinical efficacy and adverse effects. Thus, due to the distinctive properties of PNPs in terms of sizes and zeta potentials, they have been regarded as a revolutionary anticancer drug administration approach to treat LC [54,55].

A significant anticancer effect with minimal toxicities was observed when taxanes were loaded with polyethylene–polylactide (PEG–PLA) and studied in in vitro and in vivo studies [54]. Similarly, when paclitaxel and cisplatin were loaded into PEG–PLA copolymers, an excellent anticancer effect was offered. Based on the outcome, the Phase I clinical trial was successfully completed, and the Phase II clinical trial (Genexol-PM) was initiated among NSCLC patients [56]. In another study, the PEG–PLA copolymer was used to load gemcitabine for oral drug delivery, and the developed nanoformulation is currently undergoing a Phase II clinical trial [57]. Recently, polycaprolactone (PCL)- and chitosan-loaded mucoadhesive nanoformulations were developed for lung-targeted drug delivery [12]. When the docetaxel nanoparticle was compared with Taxotere (injectable docetaxel), a superior anticancer potential of the docetaxel nanoparticle was found [58]. Cisplatin and doxorubicin are extensively used in the treatment for LC, but these drugs’ side effects are limiting factors for their use. To overcome this problem, cisplatin and doxorubicin were loaded into gelatin and poly (isobutyl cyanoacrylate polymers, and the developed nanofabrication showed a potent antitumor effect with minimal toxicity [59]. In one study, hyaluronic acid in conjugation with cisplatin NP was explored in an in vivo study. Even when administered intravenously, the outcome showed a more significant antitumor effect than the conventional formulation [60]. Additionally, the outcome of the study showed minimal neurotoxicity and nephrotoxicity [60].

### 4.2. Liposome

Liposomes are bi-layered phospholipid nanocarriers and are classified as either unilamellar or multilamellar vesicles [61]. Unilamellar vesicles consist of a single bilayer, whereas multilamellar vesicles are composed of multilamellar vesicles. The size of the liposome varies from 1 to 100 nm and possesses the property of incorporating both lipophilic and hydrophilic drugs; hence, the therapeutic efficacy of the formulation is enhanced [61]. The stability, drug loading capacity, and release pattern of liposomes depend on the size and the number of the lipidic bilayer. Considering liposome-mediated pulmonary drug delivery, the use of phospholipid and cholesterol are considered as most effective and biocompatible [61].

Additionally, liposome-mediated drug delivery has been studied to overcome the problem of drug resistance and reduce side effects [62]. One of the advantages of liposomes is that the surface of the liposome can be modified, and hence, desirable targeted drug delivery can be achieved. Thus, among the various NPs, liposomes are considered as the most successful carrier system for the lungs [62]. Many of the United Food and Drug Administration (USFDA)-approved liposomal drugs are commercially available on the market. As an amphiphilic carrier system, hydrophilic drugs, such as doxorubicin and paclitaxel, can be easily loaded into a liposome [63]. When etoposide and docetaxel were incorporated into liposomes and tested for the anticancer potential in lung cancer, a significant synergistic pro-apoptotic activity via enhanced p53 activity was found [64]. A paclitaxel liposome was developed, and when pharmacokinetic profiling was done after nebulization, the area under the curve (AUC) of the nanoformulation was found to be twenty-fold higher than the paclitaxel administered via the intravenous route [65]. A significant reduction in tumor mass was found when this paclitaxel liposomal formulation was studied for its antitumor potential. As we have previously discussed, cisplatin is an extensively used drug for LC, but nephrotoxicity and hematological toxicity often restrict its use [65]. Thus, sustained-release liposomal cisplatin was fabricated to overcome this problem, and a Phase I study is ongoing. In one interesting study, an interleukin 2 (IL-2) liposome inhalation formulation was designed, fabricated, and tested, and the outcome of the study showed no evidence of toxicity, and it was found to be safe for LC patients [66].

### 4.3. Nanoemulsion

Nanoemulsions (NEs) are one of the most extensively studied nanocarriers for various disease conditions. NEs can be formulated as water in oil or oil in water, having a particle diameter in the range of 20 to 200 nm [67]. NEs are transparent and stable and consist of hydrophilic and hydrophobic phases, surfactant, and cosurfactant. Thus, most of the hydrophilic or hydrophobic drugs can be incorporated into the NE for effective targeted delivery [67]. Additionally, NE is considered an ideal carrier system for the delivery of anticancer drugs as far as bioavailability, stability, release pattern, and targeted delivery is concerned [68]. Moreover, NE protects the drug against ultraviolet (UV)-induced degradation; microbe-induced degradation offers long-term storage and can be administered intravenously, topically, or orally [69]. Considering NE in lung cancer, various synthetic, semisynthetic, and natural drugs have been incorporated into NEs and have been studied for possible anticancer effects [50,68]. Doxorubicin is another extensively used anticancer drug, but cardiotoxicity, nephrotoxicity, and hepatotoxicity are limiting factors for its use. Thus, pH-sensitive NE was explored for the possible efficacy and toxicity mitigation. The outcome of the study showed improved effectiveness and reduced mortality among the patients [70]. Paclitaxel is another extensively used anticancer drug used to treat LC, but dose-related toxicity and pharmacokinetics limit its use. Thus, to overcome this problem, NE containing paclitaxel in conjugation with hyaluronic acid was fabricated and tested in NSCLC [71]. Docetaxel was also fabricated in oil–water emulsion in which medium-chain triglycerides were used as the oil phase. When this formulation was tested for its anticancer potential, the study’s outcome showed improved AUC, slow clearance, improved volume of distribution, and tumor necrosis (as analyzed by the histopathological study) [72]. Curcuminoids are isolated from *Curcuma longa* and have been explored for multiple pharmacological activities. To enhance the pharmacological activity of lung cancer, NEs of curcuminoids were fabricated and studied in lung cancer cell lines (H460 and A549 cells). The study’s outcome showed significant antitumor activity via reduced expression of cyclin-dependent kinase 1 (CDK1), cyclin B, increased expression of p21, p53, and cell cycle arrest at the G/M phase [6]. Curcumin is among the most explored natural bioactive compounds for use in treating different types of cancer. However, curcumin suffers from the limitation of low solubility, low bioavailability, and rapid hepatic metabolism [73]. Thus, NEs of curcumin were fabricated and explored for their possible antitumor efficacy [73]. The fabricated formulation showed enhanced entrapment efficiency and improved release pattern. Furthermore, a 7.4-fold increase in bioavailability was found as compared to conventional formulation upon oral administration [73]. The molecular mechanism involved in the anticancer potential of curcumin NE in lung adenocarcinoma was found to be a modulation of extracellular receptor kinase, cyclooxygenase-2, protein kinase C, matrix metalloproteinases, and activating transcription factor 2 (ERK 1/2, COX-2, PKC, MMPs, and ATF-2, respectively) signaling pathways [74,75]. Similarly, diferuloylmethane isolated from the turmeric, 9-bromo noscapine (a tubulin-binding alkaloid), and quercetin are natural products and possess potent antitumor activities. Despite being potent and effective molecules, these two drugs suffer from pharmacokinetic limitations. Hence, their NEs were fabricated and explored for possible anticancer effects in LC [76,77,78]. The study outcome showed an improved pharmacokinetic profile and enhanced antitumor activity via apoptosis initiation and angiogenesis inhibition [78]. Lycobetaine (LBT) is a well-known alkaloid and showed significant anticancer potential via topoisomerases I and II inhibition. However, lycobetaine has a short half-life and poor bioavailability and hence, its NE was fabricated and tested in LC [79]. Danshen, tanshinones, and *Brucea javanica* oil are well-known Chinese herbs and possess potent anticancer potential. However, to enhance their pharmacological and pharmaceutical potentials, their NEs were formulated and studied in in vitro and in vivo setups [80,81,82,83].

### 4.4. Polymeric Micelle

Polymeric micelles (PMs) are biodegradable and biocompatible nanocarriers that have shown great potential for targeted drug delivery of chemotherapeutic drugs in LC [84]. They are self-assembled amphiphilic NPs that become aggregated in the presence of copolymers and solvents [84]. A wide variety of polymers are available for PM fabrication, and the choice of these polymers depends upon compatibility with the selected drugs to allow incorporation, desired loading capacity, and stability. The cores of the PMs are hydrophobic, and poorly soluble drugs are generally incorporated into these micelles [85]. PMs offer the advantages of prolonging circulation time, bypassing hepatic metabolism, and offering an improved volume of distribution. PM sizes vary from 20 to 200 nm; hence, they can easily travel through the tumor microenvironment and escape from the reticuloendothelial system (RES), usually found in the liver, spleen, kidney, lymph nodes, and bone marrow cells [85]. A large number of polymers, such as poly (styrene-co-maleic anhydride [SMA]), poly(ethylene glycol)-block-poly(d-l-lactic acid [PEG-b-PLA]), poly(ethylene glycol)-block-poly(d,l-lactic-co-glycolic-acid [PEGb-PLGA]), poly(ethylene-glycol)-block-poly(ℇ-caprolactone [PEGePCL]), poly(*N*-vinylpyrrolidone)-block-poly(ℇ-caprolactone [PVP-b-PCL]), pluronic, D-a-tocopheryl polyethylene glycol and PEG-poly(amino acid [PEGePAA]) have been studied for targeted delivery of chemotherapeutic drugs into the LC tumor [84]. SMA is a synthetic copolymer composed of maleic acid and albumin. SMA is advantageous as it is stable in the body fluid and is non-toxic [86]. SMA conjugated to neocarzinostatin was explored for the anticancer effect in lung cancer, while SMA conjugated to paclitaxel was studied for the anticancer potential against adenocarcinoma [87]. PEG-b-PLA is an FDA-approved excipient and is used for the encapsulation of various anticancer drugs [88]. PEG-b-PLA is a copolymer consisting of PLA and PEG and offers excellent properties for the encapsulation of anticancer drugs. The ratio of PLA and PEG determines the release rate and pattern of encapsulated drugs, in which low molecular weight structures showed rapid release pattern whereas high molecular weight showed a delayed release pattern [88,89,90]. Recently, paclitaxel, curcumin, and rapamycin encapsulated polymeric are being investigated for their possible effect against lung cancer. The FDA also approves PEG-b-PLGA, and it is a biodegradable polymer [91]. Similar to PEG-b-PLA, the release pattern of the drug from PEG-b-PLGA can be modulated via a change in the ratio of PEG, glycolide, and lactide [91]. PEG-b-PLGA loaded with paclitaxel and doxorubicin was studied in NSCLC. The outcome of the study showed improved and synergistic antitumor potential of these two drugs along with minimal side effects [92]. Similarly, paclitaxel and cisplatin were also encapsulated and studied for the synergistic anticancer effect in combination with radiotherapy [93].

## 5. The Limitations of Nanocarrier Drug Delivery Systems and miRNA as Emerging Tools against Lung Cancer

Currently, a lot of research is going into the development and delivery of safe and effective nano carrier-based systems targeting LC, but most of these drugs failed in clinical trials [94]. Some of the investigated reasons for the failure appear to be a lack of precise mechanism of action, toxicity due to excipient or particle size, and higher retention times in the circulatory system [53]. Thus, looking into the potential of nanocarriers against lung cancer and negative outcome in the clinical trials, researchers are now using FDA-approved excipients. Additionally, the nanocarriers have to cross numerous barriers and obstacles, such as dense matrix, protein-corona effect, phagocytosis, and drug efflux proteins before reaching the site of action [1]. Moreover, different types of tumor microenvironments respond differently to the same nanocarriers, which is one of the major issues [1]. Recently, Doxil has been reported to accumulate in Kaposi sarcoma, related to acquired immunodeficiency syndrome (AIDS), and this issue is a major area of concern for clinicians [95]. Additionally, a deep understanding of nanomedicine in the tumor microenvironment is lacking because of the unavailability of reliable preclinical models. Although xenograft models are currently being used, the findings from these models differ significantly from the human tumor microenvironment [95]. Thus, to overcome these challenges, microRNA (miRNA)-based therapeutics are being explored as possible therapeutic tools in LC [96].

## 6. miRNAs and Lung Cancer

**Figure 5:**
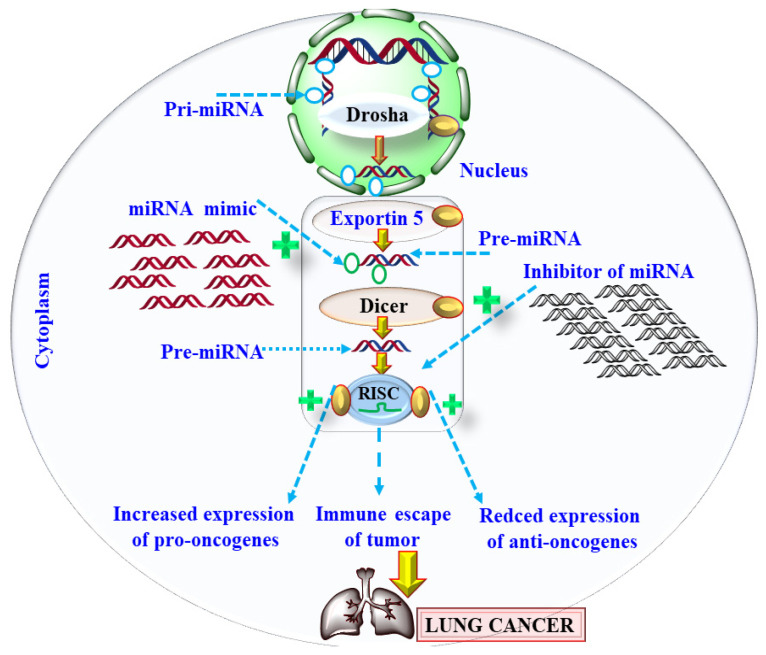
Showing the biosynthesis and the role of miRNA in lung cancer.

Currently, many chemotherapeutics are being explored for their possible anticancer effects, but most of them suffer from pharmacokinetic limitations and exhibit significant toxic effects [47]. Thus, a nano carrier-based drug delivery system was explored to address this limitation, but unfortunately, a lacuna in the desired therapeutic effect still exists. Thus, recently the role of miRNA is under investigation for its possible application in the management and treatment of LC [96]. RNA polymerase II was found to be responsible for the transcription of miRNA or pri-miRNA, upon which ribonuclease Drosha further acts, and pri-miRNA is converted into pre-miRNA [97]. This process occurs in the nucleus, and once pre-miRNA is formed, it moves out of the nucleus and into the cytoplasm, in which it is cleaved and mature miRNA is produced as shown in Figure 5 [97]. The mature mRNA gets incorporated or loaded into RNA-induced silencing complex (RISC) and argonaute. Finally, these miRNAs are involved in the silencing of mRNA. The miRNA usually binds with the complementary sequence of mRNA at three prime ends and inhibits the translation process [97]. It was further found that a single miRNA regulates the function of multiple mRNAs in humans. More than 50% of genes involved in LC are associated with miRNA [98]. Thus, miRNA is considered an emerging pathogenic factor in LC etiology and has emerged as a clinically relevant tool for managing and treating LC [99]. Based on the involvement of miRNA in carcinogenesis, miRNAs are classified as oncomiRs and tumor suppressor miRNA [99]. As the name suggests, oncomiRs are responsible for overexpression of pro-oncogenes or suppression of tumor suppressor genes, and are consistently found to be overexpressed in the tumor cells [96]. Tumor-suppressor miRNA suppresses the translatory activity of mRNA that is responsible for oncogene transcription. Thus, oncomiRs cause tumor initiation, progression, angiogenesis, invasion, and metastasis [96].

### 6.1. Mechanism of miRNA Deregulation in Lung Cancer

Involvement of miRNA in pathogenesis has been well established, and it was found that the factors that affect the biosynthesis of miRNA at the pri- or pre-miRNA level are primarily responsible for causing dysregulated miRNA and carcinogenesis [100]. Recently, p53, c-Myc, and E2F were identified as the transcription factors responsible for increased oncomiRs and reduced tumor suppressor miRNA expression [101,102]. Apart from the role of these transcription factors, epigenetic malfunction was also found to be an important factor in the increased level of dysregulated miRNA. Studies have suggested the role of hypo or hypermethylation and alterations in histone acetylation [103]. CpG methylation was studied for the increased expression of miR-223 that leads to acute myeloid leukemia [104]. Similarly, methylation of DNA and histone deacetylation were associated with dysregulated miRNA in bladder cancer [105]. Additionally, reduced expression of miRNA-148a and miR-34b and their associations with carcinogenesis were found to be associated with methylation of CpG [106].

**Figure 6:**
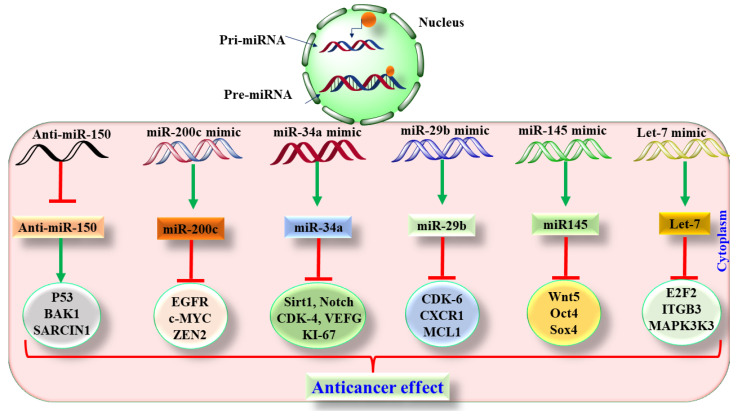
Role of various miRNA in lung cancer.

miR-29b was found to be reduced in NSCLC, whereas miR-29b was found to increase the sensitivity of cisplatin in LC [107]. The epithelial–mesenchymal transition (EMT) is one of the critical steps in tumor metastasis, and recently, miR-101, miR-200, miR-27, miR15b, and miR-451 were found to be suppressed and involved in EMT in LC [108,109,110]. Similarly, miR-17-92, miR21, miR-16, miR-200c, miR-34, and miR-29b were found to be overexpressed in LC and act as oncomiRs [111]. In LC, miR-21, and phosphatase and tensin homolog deleted on chromosome 10 (PTEN) were found to be downregulated and positively correlated with chemoresistance against TKIs. However, selectively targeting miR-21 and PTEN can be used to chemo-sensitize cisplatin in NSCLC [112]. miR-34 (a-c) has been extensively explored for involvement in the cell cycle progression via modulation of c-Myc, Bcl-2, sirtuin-1, forkhead box P1 (FOXP1), and histone deacetylase (HDACs). Among these subtypes of miRNAs, miR-34c was found to be down-regulated in LC [113,114]. miR-212 and miR-350 have been reported as tumor suppressor miRNAs that exhibit antitumor effects in LC via TRAIL-mediated apoptosis [111]. Thus, looking into the therapeutic involvement of miRNA in LC, two therapeutic approaches are currently used: (1) inhibition or blockage of oncomiRs and (2) stimulation of tumor suppressor miRNA. Various carriers, such as small molecules, oligonucleotides, or viruses, are currently being used to target the various miRNA as shown in Figure 6.

### 6.2. Preclinical Based Evidence of miRNA in Lung Cancer

Generally, for targeting oncomiRs, antisense anti-miR oligonucleotides (AMO) or locked nucleic acid (LNA), miRNA sponges, or miRNA antagomirs are used. AMO is synthetic antisense complementary to the targeted miRNA [96]. AMO binds to the miRNA and inhibits its interaction with the mRNA so that the translation of oncogenic proteins is inhibited and the mRNA performs its normal functions [115]. AMO is thermally unstable and has poor aqueous solubility [116]. Hence, LNA with improved thermal stability and enhanced aqueous solubility was developed. The use of LNA has been reported to silence miR-21 and results in increased apoptosis and reduced tumor burden [117]. Similar to LNA, antagomirs and miRNA sponges have been explored to silence the oncomiRs [118]. Apart from miRNA inhibition, restoration of miRNA is also an important therapeutic approach for treatment and management of LC. Generally, miRNA mimics or viral vectors (lentivirus, adenovirus, and retrovirus) are responsible for the miRNA expression (miR-15 and let-7) and are used to restore the normal activity of miRNA functionally [118]. Considering LC, miR-34, 29b, 20c, 145, and let-7 are tumor suppressor miRNAs, and their levels were found to be downregulated in LC [111]. To restore the normal functioning of tumor suppressor miRNA, H460/A549 cells for NSCLC were treated with the let-7 mimic, and the outcome of the study showed a significant antitumor effect [113,119]. Based on the outcome of this study, let-7 was dissolved in lipid base. It was further evaluated in a xenograft model, and a significant reduction in tumor volume was observed [119]. As we have already discussed, miR-34a is downregulated in cancer; hence, synthetic miR-34a was formulated in a lipid-based vehicle and administered to the NSCLC mice. Surprisingly, the use of this lipid-based miRNA-34s caused an effective reduction in cancer severity [113]. The observed mechanism involved in the anticancer effect was found to be reduced expression of ki-67, CDK4, and Bcl-2 [113]. Additionally, the use of this mimic was found to be safe as no sign of toxicity was observed in liver, kidney, and heart [113]. In one interesting studies, miR-145 was administered intratumorally in a lung adenocarcinoma model of mice by incorporating it into the biodegradable polyurethane-branched polyethyleneimine [120]. The study’s outcome showed EMT inhibition, increased apoptosis, reduced tumor growth, and angiogenesis [120]. Similar to miR-34a, miR-29b is also a tumor suppressor gene, and in the absence of the normal functioning of miR-29b, CDK-6 is activated and regulates the cascade of tumorigenesis [111]. Thus, a cationic-carrier-based miRNA was developed to incorporate mir-29b and administered to the A549 xenograft mice model to yield a significant antitumor effect [111].

### 6.3. Translatory and Clinical-Based Evidence of miRNA in Lung Cancer

After looking into the potent role of miRNA in the pathogenesis of LC and various preclinical studies reported so far, pharmaceutical industries have come forward to initiate studies for therapeutic implications. Recently, LNA for targeting miR-122 (SPC3649) was developed by Santaris Pharma [121,122]. This anti-miRNA was the one that was entered into a clinical trial [123,124]. MRX34 is a miR-34a mimic, and its efficacy in NSCLC has been investigated in a Phase I clinical trial (NCT01829971) with the concept of miRNA replacement therapy [125,126]. Another clinical trial (NCT01829971) has shown absolute safety, efficacy, and tolerability of this compound. Based on the outcome of these trials, Phase I (NCT02862145) was continued, and a Phase II trial was designed [111]. However, in 2016, severe immunotherapeutic adverse effects (cytokine syndrome) were reported, and this study was terminated [111]. Similarly, another ongoing trial (NCT02862145) involving MRX34 in melanoma was stopped due to unwanted side effects. Apart from MRX34, MesomiR-1 has been entered into a Phase I clinical trial (NCT02369198) for treating NSCLC [111].

## 7. Challenges in Developing miRNA-Based Therapeutics

In recent years, miRNA-based therapy has gained significant attention for LC management and treatment. Indeed, miRNA-based therapy offers several advantages over conventional and target-based therapy, however some hurdles still need to be overcome [127]. One of the major hurdles is successful penetration by the oncomiRs or tumor suppressor miRNA into the tumor cells [128]. Tumor cells consist of an extensive vascular network and a complex leaky surface that significantly alters miRNA penetration into the tumor [128]. Another major challenge for successful miRNA delivery is maintaining their stability and integrity in the systemic circulation. When an miRNA enters the systemic circulation, miRNA is degraded immediately by various RNAases and eventually cleared from the circulation [127].

Additionally, administered miRNA is also excreted via renal excretion [129]. Apart from renal clearance, fast hepatic metabolism, RE and splenic Kuffer’s cell-mediated uptake and phagocytosis via the phagosome are other barriers limiting miRNA-based therapeutic outcomes [129]. Apart from these discussed limitations, miRNAs have also been reported to induce immunotoxicity. This limitation occurs because when miRNAs are administered, the innate immune system undergoes activation and causes immunotoxicity in which interferons or Toll-like receptors (INFs or TLRs) are activated [130]. Importantly, miRNA has been reported to cause off-target silencing of various genes and results in unwanted side effects [111,130].

### Nanocarrier-Based miRNA Delivery in Lung Cancer

As we have already discussed, despite the therapeutic potential of miRNA, this system suffers from major pharmacokinetic limitations and exhibits immunotoxicity and off-target gene silencing. Thus, to overcome these limitations, nanocarrier-based miRNA (NC-miR) delivery has been used for the selective targeting of lung cancer cells [131]. When NC-miR delivery is used for lung cancer, several factors, such as tumor vasculature, interstitial fluid pressure, extracellular matrix composition, and lymphatic drainage are taken into consideration [131]. One of the most extensively used NC for the delivery of miRNA is polymeric nanoparticles [132].

Polymeric nanocarriers have also been studied for targeted delivery of miRNA in LC [133]. PEI, LGA, and poly(amidoamine [PAMAM]) are some of the well-studied cationic synthetic polymers [134]. These polymers are advantageous in terms of enhanced stability, cellular specificity, cellular uptake, a low toxicity profile, and being non-immunogenic [134]. PEI was recently used to successfully deliver miR-145 and miR-33a in a xenograft model of colon cancer. The outcome of this study showed an increase in apoptosis and a reduction in tumor growth [134]. miR-154 in combination with cisplatin encapsulated in polyurethane–polyethyleneimine was also studied in LC [135]. In one interesting study, polyarginine-disulfide in conjugation with PEI was studied for the targeted delivery of miR-145 in prostate cancer [136]. A disulphide linker was used to enhance biocompatibility and exhibit desired cytotoxic effects [136]. The study’s outcome showed a significant reduction in the rate of tumor growth and increase in the duration of survival. Poly(L-lysine) and polyethyleneimine were used to successfully deliver anti-miR-21 in breast cancer, whereas miR-145 was delivered via polyurethane conjugated with PEI for the treatment of LC [135,137]. Apart from the aforementioned polymeric nanocarriers, *N*-(3-aminopropyl) methacrylamide (APM), ethylene glycol dimethacrylate (EGDMA), and acrylamide (AAM) have been used for the delivery of miRNA, such as anti-miR-21 [138,139]. Gemcitabine is one potent anti-cancer drug, but it suffers from the limitation of chemoresistance. Recent findings have demonstrated the chemosensitizing property of miR-205; hence, a PEG and polypropylene carbonate copolymer nanocarrier was used for delivery of miR-205 in pancreatic cancer [140]. The study’s outcome showed a reduction in resistance, tumor size, growth, and weight, and caused an effective reversal of metastasis and tumor invasion [140]. Similarly, a poly(Ԑ-caprolactone [PCL]) and PEG nanocarrier was used for the delivery of miR-200c and docetaxel, and the outcome was studied in both in vitro and in vivo studies [141]. PLGA is another FDA-approved polymer with an established safety profile for miRNA drug delivery [142]. PLGA offers the advantage of surface modification and multiple ligand targeting [142]. When miR-221 was encapsulated into PLGA, increased apoptosis, reduced tumor growth, migration, angiogenesis and invasion was observed in lung and hepatic carcinoma [143]. Moreover, when PLGA in combination with PEF was studied for miR-10b in addition to anti-miR-21 delivery, a significant reduction in the rate of tumor growth was observed in breast cancer [144]. Additionally, PLGA in combination with PEI was used for co-delivery of doxorubicin and miR-542-3p. The outcome of the study showed enhanced loading capacity, increased drug uptake, cytotoxicity, and significant anti-tumor effects [145].

Dendrimers are branched polymers with the presence of an amine branch that acts as a proton sponge and helps in endosomal escape. Dendrimers have been extensively used for the targeted delivery of miRNA in various types of cancer. Poly-amidoamine (PAMAM) is a cationic polymer and one of the commonly used dendrimers used for the delivery of miRNA [146]. Recently, PAMAM was used as a nanocarrier for the targeted delivery of miR-21 in brain tumors, and the outcome of the study showed an increase in apoptosis and reduction in the rate of tumor growth [146]. Similarly, codelivery of miR-205 and anti-miR-221 using PAMAM showed a significant reduction in tumor size and an increase in survival [147].

It is also important to highlight that various natural polymers, such as chitosan and peptides, have also been studied to deliver miRNA [139]. Chitosan is extensively studied in natural polymers and reported to be biocompatible, safe, and biodegradable [139]. Chitosan and hyaluronic acid nano-complexes were used to incorporate miR-34a and doxorubicin and miR-145 in breast cancer, and the outcome of the study showed synergistic antitumor effects [148,149]. A self-assembly noncomplex was prepared by using protamine sulfate and hyaluronic acid and successfully incorporated miR-34a for the targeted delivery in breast cancer [150]. Similarly, aptamer-conjugated atelocollagen loaded miR-15a and miR16-1 (tumor suppressor miRNA) was used for targeted delivery in prostate cancer [151].

Apart from PNPs, lipid-based nanocarriers for the targeted delivery of miRNA in LC have been extensively studied. Currently, cationic, anionic, and neutral lipid-based nanocarriers (liposomes) have been studied. Liposomes easily cross the cell membrane and release the miRNA inside the cells. However, liposomes suffer from low selectivity and specificity; hence, surface modification techniques have been used to overcome these limitations [152]. Cationic liposomes are more often used for miRNA delivery because of their enhanced cell membrane affinity [107]. They are comparatively easy in terms of production and are considered safe, non-immunogenic, and non-pathogenic. In LC, reduced miR-29b was shown to be positively correlative with pathogenesis. Thus, when a cationic liposome-encapsulated with miR-29b was used, a significant reduction in tumor growth rate was observed [107]. Similarly, the administration of cationic liposomes encapsulated with miR-107 yielded a significant anti-tumor effect [153]. Based on successful preclinical and clinical reports of cationic liposomes, several products such as Lipofectamine^®^, TransIT^®^ 2020, and Oligofectamine™ are now commercially available [154]. Despite being a potent nanocarrier for miRNA delivery, liposome use is limited because of low stability and nonspecific binding affinity toward serum proteins. Hence, to overcome these limitations, polymers, such as PEG, have been conjugated to enhance their stability and half-life [155]. Moreover, liposomes offer the advantage of synergistic drug delivery of chemotherapeutic drugs and miRNA. Recently, cisplatin in combination with miR-375 has been successfully delivered using liposomes in lung cancer [89]. Liposome-based miR-34a and miR-200c have been studied for possible anticancer effects in LC, and the study outcome’s shows promising anticancer effects [156]. Additionally, miR-135a-loaded cationic immunoliposomes was also explored in cancer therapy [154].

For a long time, inorganic components have been used to fabricate nanocarriers, keeping the size and morphology as the top priority. Inorganic materials are non-toxic, non-irritating, biocompatible, and easy to synthesize. Among various inorganic nanocarriers, gold nanoparticles (AuNP) have been extensively used for the targeted delivery of miRNA in various types of cancer [157]. AuNPs are advantageous in terms of shape, size, biocompatibility, physio-chemical properties, surface functionalization, and amphiphilicity [139]. AuNP encapsulated with miR-205 was used to treat prostate cancer in PC-3 cell lines in which the administration of nanocarriers showed enhanced apoptosis in addition to reduced proliferation and rates of tumor growth [139]. It was found that the presence of miR-20a is associated with a pro-oncogenic role and protects the tumor cells against doxorubicin-induced cytotoxicity [139]. Thus, cysteamine-functionalized AuNP was used for the delivery of miR-31 that acted as a suppressor of miR-20a and exhibited a significant anti-tumour effect [158]. It was further found that AuNP showed a 10–20-fold increase in concentrations of miR-31 and miR-1323 as compared to the conventional delivery in neuroblastoma and ovarian cell lines [139]. Additionally, when thiolated AuNP was used to deliver miR-145, a significant anti-tumor effect was observed in prostrate and breast cancer [159].

Silica is one of the extensively used inorganic materials and has also been successfully used to fabricate nanocarriers for the targeted delivery of miRNA in various types of cancer [160]. Mesoporous silica nanoparticles (MSN) are silica-based inorganic nanocarriers that offer the advantage of safe, biocompatible, stable, and greater surface loading of miRNA [160]. MSN was successfully used for the delivery of miRNA-34, which is a tumor suppressor for miRNA [161]. The use of MSN-loaded miR-34 showed an increase in apoptosis and reduction in tumor growth in tumor cells. Recently, an immunoliposome loaded with PD-L1 antibody and miR-10a was tested in a cancer model, and the outcome of the study showed the significant anticancer potential of this nanocarrier system [139]. MSN was also used for the delivery of temozolomide and anti-miR-221, which eventually resulted in inhibition of the cell cycle, proliferation, and stimulated apoptosis, and overcame the issue of drug resistance [162].

Recently, magnetic compounds were also used for the targeted drug delivery of miRNA in cancer. In one study, zinc–iron oxide loaded with lethal-7a miRNA was used to treat cancer [163]. Similarly, lanthanide Ce3/4+ cations combined with oxidized PEI were used to deliver antisense miR-486, anti-miR-99a, and anti-miR-21 into human CMK leukemia and pancreatic cells [164]. Apart from MSN, carbonate apatite has been studied for the possible nanocarrier property for miRNAs. In one of the studies, miR-4689 was incorporated into carbonate apatite NP to target KRAS in addition to AKT in cancer cells [165]. Similarly, miR-29b-1-5p was also incorporated into carbonate apatite NP against Caco cell lines with confirmed KRAS mutation. The outcome of the study showed increased apoptosis, reduced proliferation, and a better safety profile [166].

## 8. Conclusions and Future Prospects

LC is one of the major causes of morbidity and mortality worldwide. The etiology of LC has been identified as multifactorial in origin [167,168]. Various signaling pathways and molecules are involved in the initiation, progression, angiogenesis, and invasion of LC [14]. Many conventional and signaling molecular-based targeted drugs have been approved by the FDA, and many more are in the pipeline. Undoubtedly, the clinical use of these approved drugs has contributed significantly to increasing progression-free survival and improved patients’ quality of life [14]. However, most of these drugs suffer from pharmacokinetic limitations of low solubility, low bioavailability, and fast hepatic metabolism, which are not capable of reaching the target site, or penetration across the tumor cell [47]. Not only this, but most of the approved drugs also suffer from the pharmacodynamic limitation of severe adverse effects when used through oral and intravenous routes [47]. In order to overcome these limitations, inhalation and intratumoral routes were used, but unfortunately, these approaches were also not up to the expectation [169,170]. Thus, nanocarrier-based targeted drug delivery was used in which many of the approved drugs were encapsulated into the suitable nanocarrier to minimize pharmacokinetics and dose-related adverse effects [53]. Additionally, nanocarrier-based drug delivery increases stability, avoids fast hepatic metabolism, and ensures the maximum drug concentration at the site of action [53]. A large number of preclinical and clinical studies have confirmed the therapeutic utility of nanocarrier-based drug delivery in LC [53]. Currently, a few clinical trials are being conducted so that more and more patients can benefit from this therapeutic approach [170].

It is of further importance to understand that the epithelial tissue of the lungs is the center of origin, and inhalation-based therapy has access to this area. However, NSCLC or SCLC can originate from any part of the lungs, such as bronchial epithelium, peripheral bronchioles, or alveolar epithelium [171]. Thus, a nanocarrier for the treatment of SCLC or NSCLC must reach a specific area for exerting a desired pharmacological effect. For example, NC with particles size of 5 to 10 μm can reach the central epithelium [171]. However, a particle size in the range of 0.1 to 3 μm is needed for crossing the deep pulmonary tissue. Moreover, an ideal nanocarrier must exhibit a sustained drug release profile, and for the treatment of stage IV lung cancer, systemic absorption is desirable. At this stage, tumor cells gain access to lymph nodes or vital organs [172]. Thus, with the same nanocarrier system, systemic in addition to localized absorption of the drug is challenging. Thus, extensive research concluded that a multilamellar liposome is the best option for localized absorption, whereas dendrimers are suitable for systemic absorption [173].

Another problem encountered in using nanocarriers in LC is the later stage of diagnosis. Presuming that it is diagnosed at the early stage, patients also have difficulty in breathing, breathlessness, lower tidal volume, and total lung capacity. In such cases, drug delivery and absorption from the peripheral tissue are difficult [172]. Hence, spacers and power sources were used to deliver drugs at the nano size so that they could be absorbed in the deep tissue. Despite being a novel and promising therapeutic approach, nanocarrier-based drug delivery has limitations, such as toxicity due to multiple components, phagocytosis, and drug efflux ineffectiveness due to complex vasculature and the inability to penetrate the tumor mass [174]. Hence, recently, miRNA has been identified as an emerging weapon against lung cancer. However, the use of necked miRNA was associated with fast degradation by RNAse, problems in crossing biological membranes, rapid clearance, and thermal instability when administered [99]. Hence, techniques, such as chemical modification, encapsulating them in suitable nanocarriers, and using cationic polymers, have been used to overcome these limitations [175]. Currently, a few nanocarrier-based (lipid-based) miRNAs, such as MRX34, miR-34a, and let-7, are under clinical investigation for possible use in LC treatment [175].

Thus, based on the in-depth literature survey, available clinical evidence, and completed clinical trials, we suggest that a safe and effective nanocarrier system should be developed for the targeted delivery of chemotherapeutic drugs in addition to miRNA. Genomic expression of mRNA in addition to pathway enrichment analysis should be done to identify selective targets for miRNA. Furthermore, to avoid the toxicity and off-targeted side effects and also achieve cell/target-specific delivery of chemotherapeutic drugs and miRNA, low dose combinations of miRNA and anticancer drugs, radiotherapy, and immunotherapy can be used. Additionally, the antibody-coated combination of miRNA and existing anticancer agents should be used in a suitable nanocarrier system. This novel drug delivery system may pave the way for clinical treatment in the coming years.

## Figures and Tables

**Table 1:** Showing the details of FDA approved drugs and their adverse effects.

Drugs	Year of Approval	Mechanism of Action	Adverse Effect	References
Afatinib	2013	EGFR tyrosine kinase inhibitor	Diarrhea, rash, mucositis, swelling of the lips, nail infection, and nose bleeds.	[48]
Alectinib	2017	EGFR tyrosine kinase inhibitor	Bloody urine, joint pain or swelling, increased blood pressure, immobility, and nephrotoxicity.	[48]
Amivantamab-vmjw	2021	EGFR tyrosine kinase inhibitor	Shortness of breath, muscle and joint pain, swelling of hands.	[48]
Atezolizumab	2020	PD-1 receptor inhibitor	Bladder pain, bloating, ear congestion and dyspnea.	[47]
Bevacizumab	2006	VEGF inhibitor	Cardiotoxicity, alopecia, xeroderma, hemorrhage, proteinuria, and necrotizing fasciitis.	[49]
Brigatinib	2020	Inhibitor of AKT, ERK, and STAT3	Headache, skin rashes, nausea, constipation and numbness.	[47]
Capmatinib	2020	MET kinase inhibitor	Loss of appetite, chest pain and bloating.	[47]
Cemiplimab-rwlc	2021	PD-1 receptor inhibitor	Blisters, immobility, gland and joint swelling and mouth ulcers.	[47]
Ceritinib	2017	ALK phosphorylation inhibitor	Reduced hemoglobin, hepatotoxicity, and nephrotoxicity.	[47]
Crizotinib	2016	RTK inhibitor	Oedema, reduced appetite, loss of taste and hepatotoxicity.	[47]
Dabrafenib	2017	BRAF and RAF kinase inhibitor	Joint pain, papilloma, alopecia, and hepatotoxicity.	[47]
Dacomitinib	2018	EGFR tyrosine kinase inhibitor	Dermatitis, acne, stomatitis, dry skin, and paronychia.	[48]
Docetaxel	2005	Microtubule depolymerization inhibition	Neutropenia, dysgeusia hypersensitivity, anemia, thrombocytopenia, anorexia, nail disorders and fluid retention.	[47]
Doxorubicin	1970	Topoisomerase II inhibitor	Cardiotoxicity, hepatotoxicity and nephrotoxicity.	[47]
Durvalumab	2020	PD-1 receptor inhibitor	Musculoskeletal pain, loss of appetite, and UTI.	[47]
Entrectinib	2019	RTK inhibitor	Peripheral edema, hepato-reno toxicity, myelotoxicity.	[47]
Erlotinib	2010	EGFR tyrosine kinase inhibitor	Fatigue, rashes, hepatotoxicity, cough, mouth ulceration, and dry skin.	[48]
Everolimus	2016	mTORC1 inhibitor	Insomnia, weight loss, and dry mouth.	[48]
Gefitinib	2015	EGFR tyrosine kinase inhibitor	Rash, diarrhea, hepatotoxicity, acne, and anorexia.	[48]
Gemcitabine	2005	DNA synthesis inhibitor	Hair loss, nausea, mouth ulcer.	[47]
Ipilimumab	2020	Inhibition of T-cell inactivation	Diarrhea, fatigue, skin rash, and pruritus.	[49]
Methotrexate	1970	Dihydrofolate reductase inhibitor	Alopecia, hepatotoxicity, and tender gums.	[47]
Necitumumab	2015	EGFR tyrosine kinase inhibitor	Weight loss, hypokalemia, mouth ulcer, acne, and chest infection.	[47]
Nivolumab	2018	PD-1 receptor inhibitor	Lymphopenia, fatigue, diarrhea, pruritus, and vitiligo.	[49]
Osimertinib	2020	EGFR tyrosine kinase inhibitor	Diarrhea, nausea, reduced appetite, dry skin, paronychia.	[48]
Paclitaxel protein-bound nanoparticle	2012	Causes cell cycle arrest	Low blood counts, alopecia, mouth ulcer, peripheral neuropathy, arthralgias, and myalgias.	[47]
Pembrolizumab	2016	PD-1 receptor inhibitor	Anemia, hypertension, hyponatremia, hypoalbuminemia, and cough.	[49]
Pemetrexed	2017	Purine and pyrimidine synthesis inhibitor	Weight loss, vomiting, fatigue, loss of appetite, and insomnia.	[49]
Pralsetinib	2020	RET kinase inhibitor	Shortness of breath, cough, bleeding gums, nosebleeds, and mental confusion.	[49]
Ramucirumab	2020	VEGF inhibitor	Cardiotoxicity, wound healing problem and skin rashes.	[49]
Selpercatinib	2020	RTK inhibitor	Dry mouth, hypertension, peripheral edema, diabetes, and hepatotoxicity.	[47]
Sotorasib	2021	KRAS G12C inhibitor	Bone/joint pain, constipation, and stomach pain.	[47]
Tepotinib	2021	Kinase inhibitor	Anxiety, tachycardia, loss of appetite, sore throat, and stomach pain.	[47]
Trametinib	2015	MEK ½ inhibitor	Losing of fingernails, eye dryness, damaged taste buds, dry skin, and canker sores.	[47]
Vinorelbine	1994	Cycle arrest via binding with microtubular spindle	Muscle or joint pain, constipation, and loss of appetite	[47]

## Data Availability

The data presented in this study are available in article.
